# Customisable IT tool for on-field assessments to support disaster management

**DOI:** 10.1038/s41598-023-47521-x

**Published:** 2023-11-29

**Authors:** Stefano Grimaz, Petra Malisan, Fabio Zorzini, Loris Grimaz, Mauro Bettuzzi

**Affiliations:** 1 SPRINT-Lab, Polytechnic Department of Engineering and Architecture, University of Udine, Udine, Italy; 2 CG Soluzioni Informatiche S.R.L., Udine, Italy

**Keywords:** Environmental impact, Natural hazards, Civil engineering, Software

## Abstract

Information and technology (IT) based tools are widely used in all phases of the disaster management cycle (DMC), improving the effectiveness of the prevision-prevention, preparedness, response, and recovery phases. The availability of on-field collected data provides an answer to the need to represent the scenario in which decision-makers will intervene. In this context, disaster management experience has shown that in many cases the users of on-field assessment outcomes (i.e., decision-makers) need: a data collection tool quickly and simply adaptable to better respond to specific and conditional needs; the automated elaboration of situation results; and the ability to exchange the information between the different phases of the DMC. This paper illustrates the IT tool, called "SPRINT-Engine", which was developed to meet these requirements. The SPRINT-Engine has been specifically developed and improved to permit easy and rapid customizability, even in near real-time. The paper presents some applications in real cases of on-field assessment managed by SPRINT-Lab researchers in support of institutions in various activities and projects at different scales and in different phases of the DMC.

## Introduction

**Figure 1:**
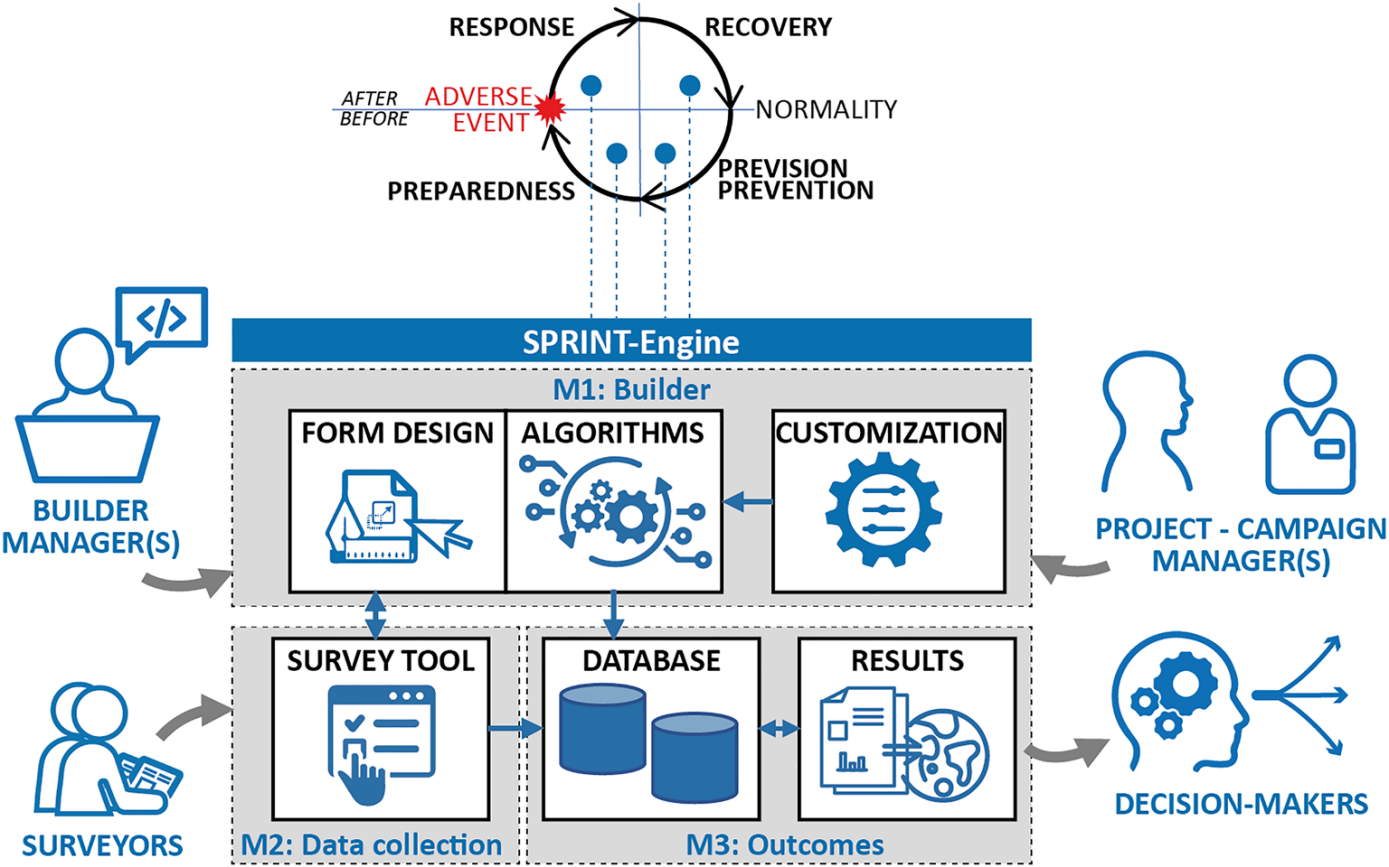
The top part illustrates the connection of the SPRINT-Engine with all the phases of the disaster management cycle. The bottom part summarises the architecture of the SPRINT-Engine, including its main modules, key subjects, and their connections.

A disaster refers to a widespread and severe disruption of the functioning of communities, ecosystems, or infrastructure due to a profound and often catastrophic event, natural or human-induced^1^. It leads to significant loss of life, property, and livelihoods, surpassing the affected region's capacity to cope using its available resources. Disasters can result from diverse origins, such as geological (earthquakes, volcanic eruptions), hydro-meteorological (hurricanes, floods), technological (nuclear accidents, industrial explosions), biological (pandemics, disease outbreaks), or man-made (conflicts, terrorist attacks) origins, sometimes combining in complex ways. Coping with a disaster requires the identification, collection, and processing of substantial (i.e., informative and essential) information to describe the situation both pre- and post-event. Once this information is available, it provides decision-makers with the necessary knowledge to make informed decisions and take action in response to the contextual instances^2^. Information-based decision-making is required in all phases of the so-called “Disaster Management Cycle” (DMC), i.e., response, recovery, prevision-prevention, and preparedness^3^ (top part of Fig. 1). The response is the first phase after an adverse event; in some cases, it can also begin while the event is still occurring. During this phase, disaster management efforts focus on minimising the effects of the adverse event, saving lives, and ensuring basic needs. Management actions have to be prompt and based on substantial information acquired in a short period of time^4–6^. While the modalities for identifying substantial information are discussed in other papers^6^, it is essential to emphasise the potential need for customising in “near real-time” both the set of substantial information and its acquisition support tools. The customisation requirement depends on the specific characteristics of the adverse event and of the response phase; the latter is usually characterized by a complex set of problems, which could also evolve quickly or change drastically, both with location and time^7,8^. Following the response phase, the recovery calls for decision-makers to identify and implement actions aimed at returning the situation to normal (or to a “new normal”, targeting an improved state according to the “build back better” principle). The prevision-prevention (also called mitigation), and preparedness phases seek to implement actions in anticipation of an adverse event. The prevision-prevention phase aims to take action to avoid the onset of a disaster, and therefore its adverse effects, or to limit or reduce the impact of an adverse event. The preparedness phase includes all actions to prepare for and deal with a disaster. Actions developed in these phases require specific information to support planning.

It is worth noting that the DMC phases are interdependent and therefore the information collected for one phase can (and should) also be used in the other phases. The management of disasters, both in the pre- and post-event phases, requires the collection of data that functionally represent the situation in which it is intended to operate, to support the identification, definition, and implementation of management actions. The data to be collected varies according to the type of event, the DMC phase, as well as the context that characterizes the area of interest, demonstrating the need for easily and quickly adaptable data collection tools.

There is a variety of IT tools to support surveys for data collection^9^, also using different technologies and approaches. In recent decades, IT tools functional to on-field data collection are increasingly used not only in the preparedness, prevision-prevention, and recovery phases but also in the response phase^10^. On one hand, these tools improve the effectiveness and efficiency of the data collection phase, but on the other hand, they require ad-hoc phases of design, development, implementation, testing, and training, functional to their creation and use. This constitutes a weak point when rapid customisation in near real-time is required.

Considering the requirements described above, this paper illustrates how the Authors have designed and created a new IT suite of tools, namely SPRINT-Engine, for the easy and rapid creation and use of customisable data-collection forms, to collect data for outlining the situation through multiple inspection points within a study area. The aim is to fulfil the need for an on-field assessment IT tool that can be used across the different DMC phases and that supports linking their outcomes. The tool has to allow for the collection of data by several surveyors scattered throughout the study area(s). It has to allow real-time application of rules and criteria during the surveys, potentially enabling surveyors to countercheck the situation evaluation on the basis of the provided data. As surveyors may have very different skills and capacities, an additional requirement is to provide for the possibility to use the tool as a knowledge transfer tool (during tests or exercises), using information acquired in past projects and allowing to use it for training new users. The SPRINT-Engine must also provide the possibility to create forms with a layout that facilitates data entry (e.g. ergonomic design). The tool may be managed also by users who do not necessarily have extensive IT programming knowledge.

The SPRINT-Engine has been developed specifically to answer to these requirements, through a specific research activity led by the Safety and Protection Intersectoral (SPRINT) Laboratory of the University of Udine (Italy). The following sections illustrate first how the SPRINT-Engine was organised and designed and then present its modular architecture with a description of each main module. In the applications section, four projects using SPRINT-Engine are briefly presented to show how the IT tool can provide support in the DMC phases. It is worth noting that the SPRINT-Engine is a self-developed tool aimed at answering the need of having a flexible and quickly customisable tool for applications of the SPRINT-Lab researchers in various projects in the context of disaster risk reduction. The tool has improved over the years by taking into consideration the needs of on-field applications and user feedback. For this reason, this paper doesn’t make comparisons with other existing tools, but it is limited to describing the architecture and applications of the SPRINT-Engine evidencing its characteristics and potentialities.

## The architecture of the SPRINT-Engine

While collaborating on-field during the various phases of the DMC with the Italian National Firefighters Corps, the National Civil Protection Department (DPC), and the Friuli Venezia Giulia Regional Civil Protection (PCR-FVG), the SPRINT-Lab researchers helped develop tools and procedures for gathering and processing information to support decision-makers^6,11–13^. The feedback from these experiences highlighted the need for an IT tool with the specifications listed in Table 1. Such a tool would simplify and speed up the on-field assessment activities and improve their effectiveness. The SPRINT-Engine has been expressly developed to accommodate these specificities, and it has been conceived to allow managers in a management room to create and distribute data collection forms to a multitude of surveyors scattered throughout the area to be surveyed (at the local, regional, or global level). Then, it allows the simple and overall representation of the outcomes in different ways, depending on the specific needs of the decision-makers.

**Table 1:** Specificities at the base of the SPRINT-Engine development.

Specificities at the base of the SPRINT-Engine development
Free layout of the form (ergonomic design)
Possibility to include images/graphics to adapt the tool to surveyor needs
Rapid and simple acquisition of photos and association to survey items
Possibility to permit multiple IT managers to remotely make changes in forms in real-time
Easy and rapid creation of forms, by an IT manager
Real-time changes of survey forms, by an IT manager
Real-time application of rules and criteria to countercheck some outcomes on-field
Possibility to implement from very simple to complicated criteria in the evaluation algorithms
Customisation module to assign different variable values depending on the campaign
Ad-hoc user roles assignment (e.g., managers, surveyors, experts, observers)
Possibility to preliminarily fill some sections of the forms during the desk work
Possibility to validate the survey data from a back-office (either by the surveyor or by an expert)
Online and offline surveys through Android devices
Surveyor's account: created and managed hierarchically
User identification: username and password
Management of multiple sub-objects
Duplication of campaigns
Possibility to use the SPRINT-Engine for capacity building/simulations
Links to other software (to create web-maps, web-albums, automatic reporting etc.)
Online safe access to the SPRINT-Engine (via browser) to manage all processes
Possibility to rapidly customise the SPRINT-Engine according to user feedback

To meet these requirements, the SPRINT-Engine has been developed through a modular architecture, that allows each module to be easily customised and quickly tailored to the survey needs and to all phases of the DMC (Fig. 1):

- *builder* module (M1), which allows managers to create customisable forms for collecting survey data and define algorithms for elaborating and representing the outcomes.
- *data collection* module (M2), an application linked to the builder module that allows surveyors to use the data collection forms both online and offline, and to elaborate in real-time information.
- *outcomes* module (M3), a set of tools to support decision-makers in analysing the outcomes of the surveys.

The following sections describe first the organisation of the key subjects working with the SPRINT-Engine tool, and then the three main modules of the SPRINT-Engine.

### Key subjects and organisation

**Figure 2:**
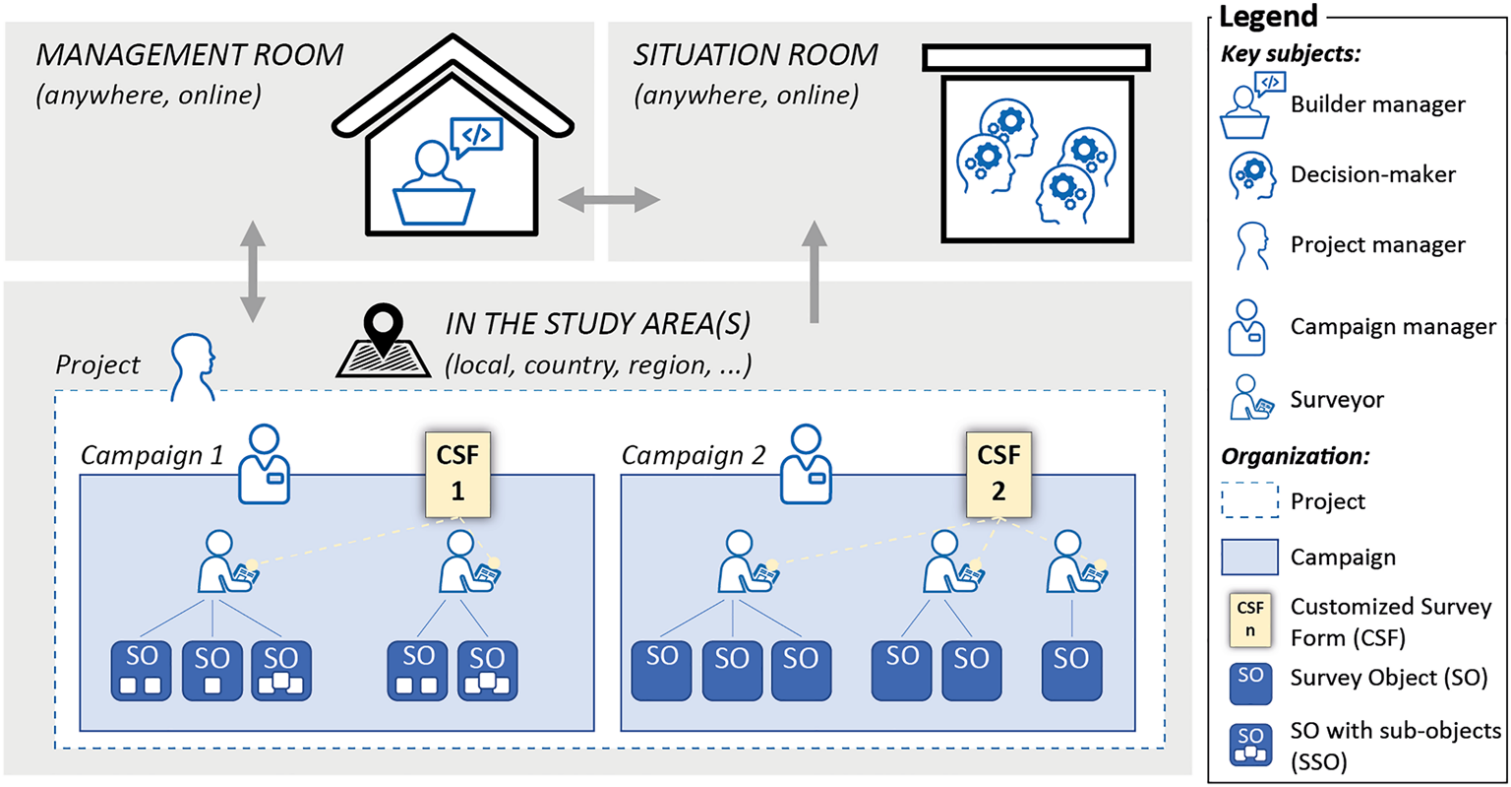
Organisation for the surveys with the SPRINT-Engine, with identification of involved key subjects.

Figure 2 illustrates the organisation for managing on-field assessment activities with the SPRINT-Engine. Key subjects, which work in close collaboration with each other, include builder managers working remotely in a management room, decision-makers in the situation room, and project managers, campaign managers, and surveyors in the study area(s).

Projects are managed by project managers, and each project can have one or more campaigns managed by campaign managers. Campaign managers identify and oversee the surveyors, responsible for on-field assessments. These key subjects are assigned hierarchically and are identified by their username, email, and password within the SPRINT-Engine.

The organisation of the SPRINT-Engine centres around campaigns, each with a specific purpose and a (potentially) customized survey form. The campaigns can be built from scratch or based on data from previous campaigns.

During a campaign, the surveyors investigate various elements known as “survey objects” (SO), which can be physical entities (e.g., buildings and infrastructures) or non-physical entities (e.g., opinions and observations). Each SO may or may not have one or more survey sub-objects (SSO). For instance, in Fig. 2, Campaign 1 SOs have one or more SSOs, while Campaign 2 SOs have no SSO.

### Builder module

The *builder* module of the SPRINT-Engine aims to create the survey forms, with the associated algorithms that determine how the collected data are processed to define the outcomes. The builder module is divided into three main sub-modules (Fig. 1): the *form design* and the *algorithms* sub-modules, which are closely related and work together, and the *customisation* sub-module, which allows customizing the algorithms to take into account local specificities.

The *form design* sub-module is used to create the layout of the data collection forms. This sub-module enables the composition of forms by inserting all necessary elements for data collection in a user-friendly manner. To create the electronic version of the form, it is first necessary to recognize and take into account the survey modalities expected for the surveys, and to determine a functional and ergonomic layout of the form, considering the skills of the surveyors.

**Figure 3:**
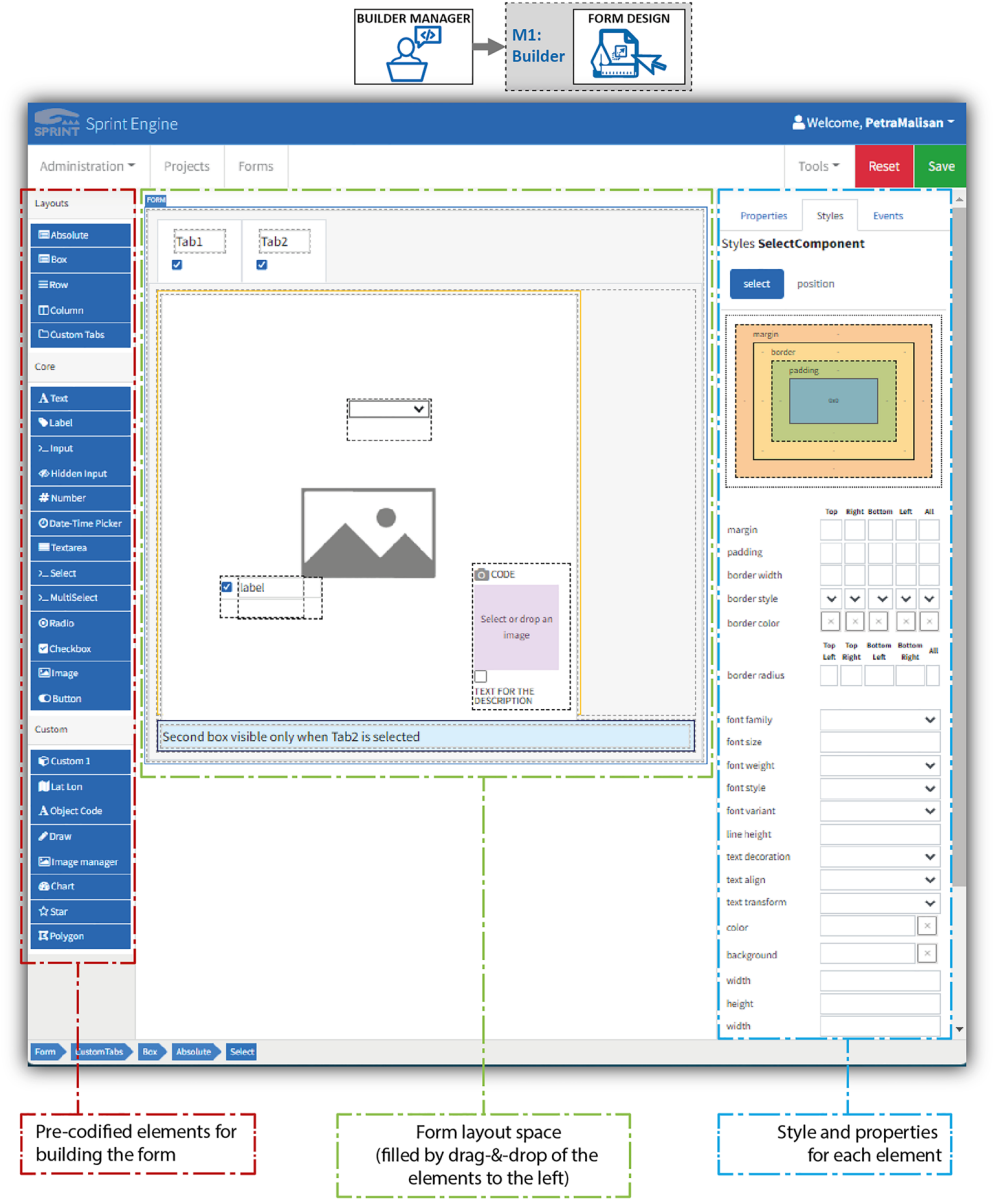
Example of form design sub-module, which is used by builder managers to create and share SPRINT-Engine forms. The dedicated front end is divided into three main sections (from left to right): pre-codified elements for building the form, form layout space (to be filled by dragging and dropping the elements), and style and properties section (for defining the characteristics of each element used in the form).

In particular, Fig. 3 shows the front end of the *form design* sub-module and highlights the three main sections: the list of the pre-coded elements for building the form, comprising typical form elements and also new elements specifically developed and implemented within the SPRINT-Engine project; the layout area of the form to be developed, which is filled in by dragging and dropping the pre-coded elements; and the style and properties to define the characteristics of each element used in the form (e.g., width, background colour, border). The layout or style of the form can also be changed dynamically, depending on the surveyors’ inputs. The simplicity of the form design tool makes it possible to quickly adapt the forms to the specific characteristics of the situation. For example, the integration of a new field for information collection into a form can be done and shared with surveyors in almost real-time.

The *algorithms* sub-module enables the builder manager to define algorithms to be directly implemented within the form. The scripts are defined using JavaScript, JQuery and CSS languages. In this sub-module, it is possible to implement very simple rules (e.g., if-else) up to very complicated algorithms (in the latter case, higher expertise is required). If needed, this sub-module also facilitates the implementation of algorithms to calculate and display the outcomes of each survey in real-time.

The *customisation* sub-module allows the adaptation of algorithms and variables for a specific use of the forms during a survey campaign, ensuring tailored results for any given situation. To facilitate this, customization forms need to be compiled by the campaign manager once for each survey campaign. This feature was developed to address the need to adapt outcomes to the local context, even when using a generic method. For example, in a project assessing the safety upgrading needs of learning facilities, the average construction cost of a new building is a variable that depends on the country where the assessment is carried out. This value can be adjusted by the campaign managers and the outcomes assessed for the campaign are calculated using the defined value.

### Data collection module

The *data collection* module provides a surveyor front-end (named SPRINT-app) that converts the form prepared with the builder module into a form that can be used in surveys through an application on IT devices. The form is converted into an HTML file with associated scripts, which is then used either on a browser website or in the SPRINT-app (currently only for Android devices). The SPRINT-app can be used both online and offline. Online access is required to synchronise the survey forms and data, but afterwards, it is possible to use the device in offline mode during the survey. All data and images are stored in a local folder and synchronised on-demand with the SPRINT-Engine database. The survey forms can also be partially filled from a computer, which is convenient for pre-survey desk work, or for post-survey review.

### Outcomes module

The *outcomes* module is divided into the *database* and *results* sub-modules. The *database* stores survey data, photos, and the calculated results. The data can then be extracted from the database in "JSON" format, which allows for greater versatility and usability of the content. Photos are extracted in a compressed file. The database can be queried online through the SPRINT-Engine web page, or with software such as Python and Matlab©, which can also be used for further processing. Through this processing, it is possible to generate specific additional results for each survey (e.g. a report with information, photos and outcomes) and for the entire survey campaign.

The *results* sub-module consists of a set of tools (e.g., reports, photo albums, web-GIS, dashboards) designed to illustrate the data and outcomes of each survey campaign and to support decision-making in the different phases of the DMC. The database of information and photos is another outcome of the SPRINT-Engine, which can be used for creating reports for a single survey and for the whole campaign.

The photos taken during the surveys can be used to build a photo database. As part of the SPRINT-Engine *outcomes* modules, a web-based repository for photos was implemented, that links the databases to the open-source photo gallery software© Piwigo^14^. This tool enables the creation of online photo albums organized according to certain specifications and allows one or more tags to be associated with each photo, for easy searching in the albums. In addition, videos can also be uploaded.

Moreover, the spatial information associated with most surveys allows the creation of web maps that dynamically display the spatial distribution of survey data and outcomes. The web maps of spatial data and associated information are created using a QGIS® server (v. 3.22) and the LizMap (v.3.6) web client rendering engine^15^. Within Lizmap, the use of the DataViz plugin also allows the display of dashboards summarising the outcomes of the campaigns. These outcomes are particularly useful when they can be made available and viewed online and in real-time in situation rooms, especially in the response phase.

## Applications

The SPRINT-Engine is used by researchers at the SPRINT-Lab to collect data in the context of different projects related to different phases of the DMC. The following sections describe the contribution of the SPRINT-Engine in four projects:

- VISUS projects (prevision-prevention, and marginally preparedness phases), aimed at supporting decision-makers in the definition of safety upgrading actions for upgrading the safety of learning facilities. VISUS stands for “Visual Inspections for defining Safety Upgrading Strategies”. Projects applied in various countries worldwide, under UNESCO’s coordination.
- COPEv project (preparedness and response phases), to support technicians in the post-earthquake operative check of (mainly) public buildings. COPEv is an Italian acronym for “Operativity Check of Buildings after a Seismic Event”. Projects developed in Friuli Venezia Giulia (Italy) with PCR-FVG.
- RSP project (preparedness and response phases), for the rapid and large-scale identification of the damage level of buildings after an earthquake by trained civil protection volunteers. RSP stands for “Procedure for Pre-codified Situational Recognition of damage evidence after a seismic event”. Projects developed in Friuli Venezia Giulia (Italy) with PCR-FVG.
- VISIVIA project (prevision-prevention phase), for the safety inspections of road infrastructures; VISIVIA is an Italian acronym for “Visual safety inspections of road infrastructures”. Projects developed in Italy, with ANSFISA (National Agency for the Safety of Railways, and Roads and Highways Infrastructures).

The following sections briefly describe how the SPRINT-Engine was used to support the implementation of the four projects.

### VISUS projects

VISUS projects are based on the VISUS methodology, a technical-triage methodology developed by the SPRINT-Lab researchers. The aim of VISUS is to define a decision support tool for planning strategies to reduce the multi-hazard risk of learning facilities^16–19^. VISUS has been adopted by UNESCO and tested positively in several pilot projects^20,21^. One of the distinctive features of the VISUS methodology is that it uses a graphical language to facilitate and speed up the collection of substantial information during inspections of learning facilities. For surveyors, the adoption of a graphical language (i.e. pictograms) is an essential support as it can summarise the information better and faster than a textual description. In VISUS, the fields represented by pictograms are called “OBS” (observables). A correct and precise interpretation of OBS is guaranteed by specific training of surveyors (supported by a photo database created with photos from past projects). The SPRINT-Engine is used to develop the VISUS survey form (called “VISUS finder”), which facilitates surveyors during the inspections of schools. In addition, the SPRINT-Engine permits the automated creation of outcomes using the assessed school safety situation and safety upgrading needs, which provide support for decision-makers in the definition of safety upgrading strategies for the schools.

**Figure 4:**
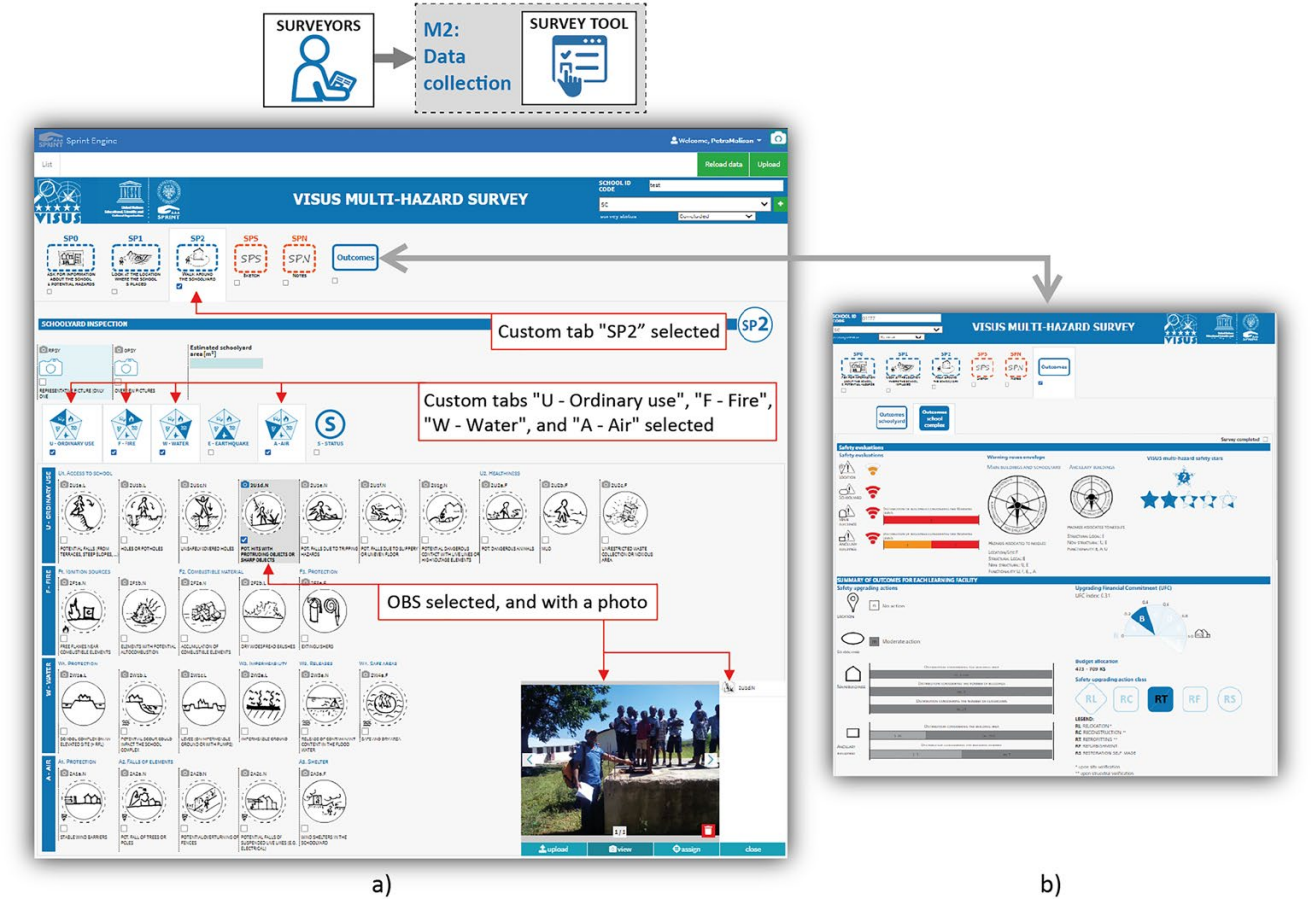
Example of survey tool in the data collection M2 module. (**a**) Example of a section of the VISUS finder form (created with M1 form design sub-module). The image shows the “SP2” custom tab, and, in sub-order, the “U—Ordinary use”, “F—Fire”, “W—Water”, and “A—Air” custom tabs selected. The OBS with the code “2U1d.N” is selected as it has one photo assigned which allows the surveyor to show where he/she identified the issue. (**b**) Example of “outcomes” section, which applies in real-time the algorithms and calculates the outcomes during the on-field assessment.

In the VISUS finder, the SO is the school complex, and the SSOs are the school buildings, the number of which can be variable in each school. The VISUS finder therefore has a specific form for inspecting the common areas of the school (e.g., location, school data, schoolyard) and a form for inspecting the individual school buildings. For the school complex and the school buildings, the VISUS finder forms are organized according to a structure that is functional to the VISUS inspections, i.e., distinguishing five survey phases (SP) and the hazards (earthquake, water-related, air-related, and fire hazards, and threats from the day-by-day use of the school) associated with each piece of information. In the VISUS finder, this structure is developed through tailored tabs, i.e., specific layout elements of the *form design* sub-module that allow the user to select for which tabs the content should be visible (Fig. 4 shows an example where only the content of tab “SP2” is visible). OBS are represented by another tailored element of the form that allows the surveyor to click on a pictogram to check a specific element (defined by a variable name or code). It is also possible to click on a small camera icon to take a picture (from the camera or gallery) and automatically link it to the element variable. Figure 4 shows many OBS elements; when an OBS element is selected, its background turns dark grey, and the small camera icon turns blue if it has an image associated with it.

Before closing the survey, outcomes are automatically calculated using the algorithms defined in the *algorithms* sub-module and the information in the customisation forms. The outcomes are presented in a specific section of the form (Fig. 4b); this aims at transferring knowledge to the surveyor, who can see how outcomes change depending on the selected items. When a survey is completed, the campaign manager can review the data, possibly edit some assignments, and validate the survey.

**Figure 5:**
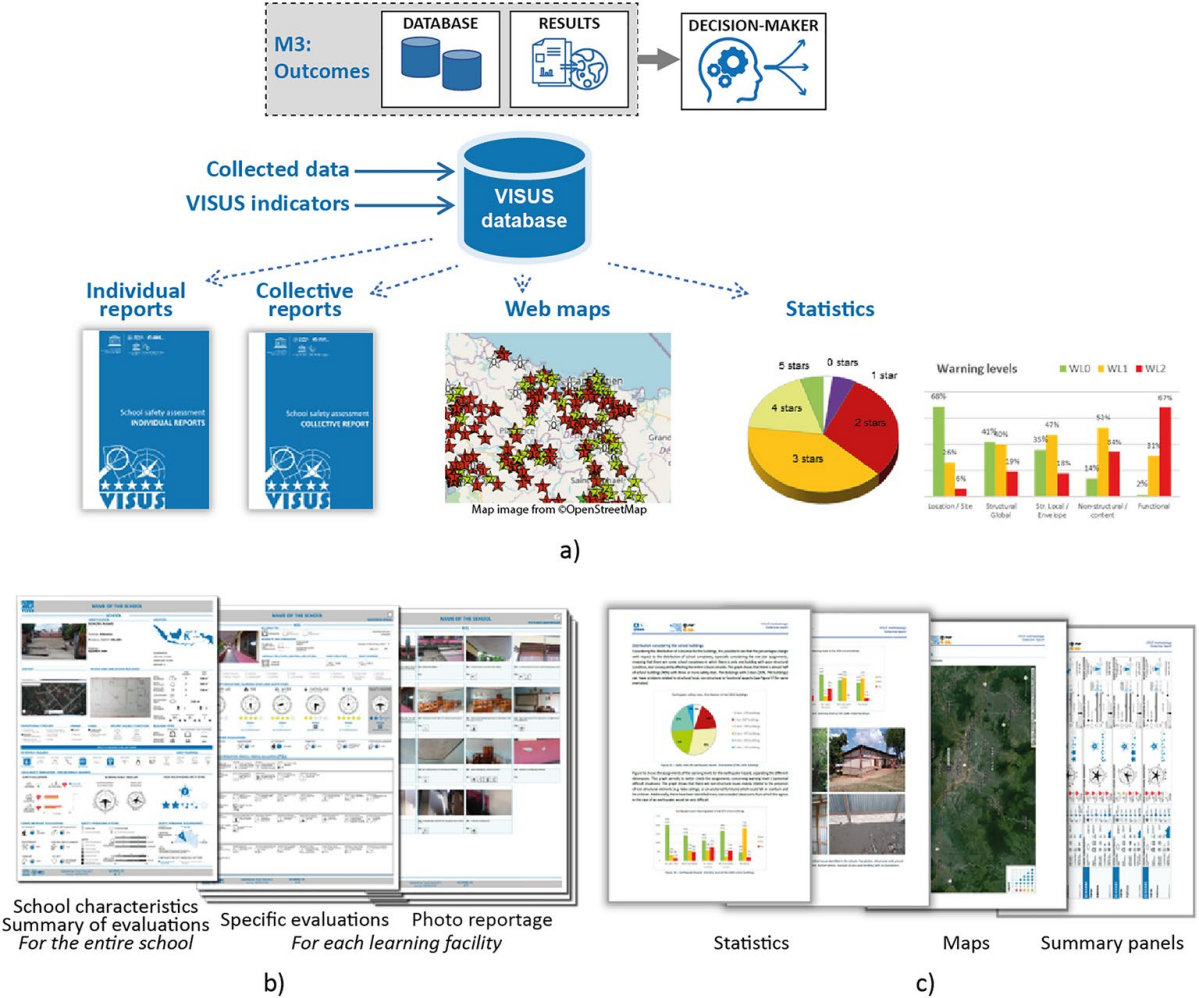
Example of outcomes obtained with the M3 SPRINT-Engine module (extract from an application in Indonesia, data intentionally made non-readable). (**a**) The outcomes include a database of survey data, photos, and VISUS indicators; individual and collective reports; a web map summarizing the outcomes (map image from ©OpenStreetMap, Web Map Service: LizMap^15^); and statistics for supporting decision-makers in planning. (**b**) An excerpt from an individual report, which includes a page with the key characteristics of the school and a summary of the outcomes, as well as two or more pages for each learning facility (buildings and schoolyard) with a technical description of the evaluations and a photo report of the inspected facility. (**c**) An excerpt from the collective report, with summary statistics of the outcomes, representative photos of issues encountered, a web map (with link), and summary panels to provide an overview of the situation.

Once the survey campaign has been completed, it is possible to extract and share the database of survey data, outcomes, and pictures. It is also possible to connect to the database online to use the data for further representations of the outcomes, such as in a web-GIS, or to automatically generate reports for each school or for the whole campaign. Figure 5 shows an example of a summary of the outcomes generated using module M3 of the SPRINT-Engine in the VISUS methodology. In addition, the photos taken during the survey are used to enhance the “VISUS photo album”, which provides surveyors with real-case examples to support the recognition of the different OBS.

The use of the SPRINT-Engine for the implementation of VISUS projects has had a positive impact both on capacity building and on the utilization of the results. Concerning capacity building, the SPRINT-Engine allows for simulations to be carried out, which have proven to strongly improve the knowledge transfer to VISUS surveyors. In this sense, post-project feedback has highlighted a significant advantage in exploring simulations as a capacity building tool compared to the previous situation when data were elaborated after the survey execution and results were not shared with surveyors. Concerning the utilization of results, decision makers’ feedback underlines the importance of having both technical reports for the individual school and collective reports and maps to oversee the entire situation.

### COPEv projects

As part of the INTERREG ARMONIA project^22^, researchers from the SPRINT-Lab, in collaboration with the PCR-FVG developed the COPEv form for the operativity check of buildings after a seismic event^23,24^. The form was first developed in a paper-based version, and then the electronic version was also created with the SPRINT-Engine.

The purpose of the form is to support technicians during the check of (mainly) public buildings after a seismic event (usually of low-moderate intensity). The check aims to identify the presence of damage that could jeopardise the resumption of regular activities in the building. The form is designed to be partially filled by technicians prior to a seismic event (i.e., during the preparedness phase), including specific technical information about the building. After the occurrence of a relatively small seismic event, if the building has not suffered severe damage, a technician is called to check the condition of non-structural elements and systems in the building, to evaluate the possibility of resuming regular activities in the building. The check is carried out on the basis of the previously filled form, which simplifies and guides the activities of the technician and supports the central management system to identify problems at the territorial level. Therefore, after an event (response phase), technicians use the form to report (also with photos) the situation and to analyse the state of the systems previously described. As the possibility of aftershocks is high, the surveyors may be called to fill out the forms multiple times to report the effects of each event. The SPRINT-Engine allows these aspects to be addressed by duplicating the campaign forms, so that, when a new event occurs, the surveyors can use the new duplicated forms.

**Figure 6:**
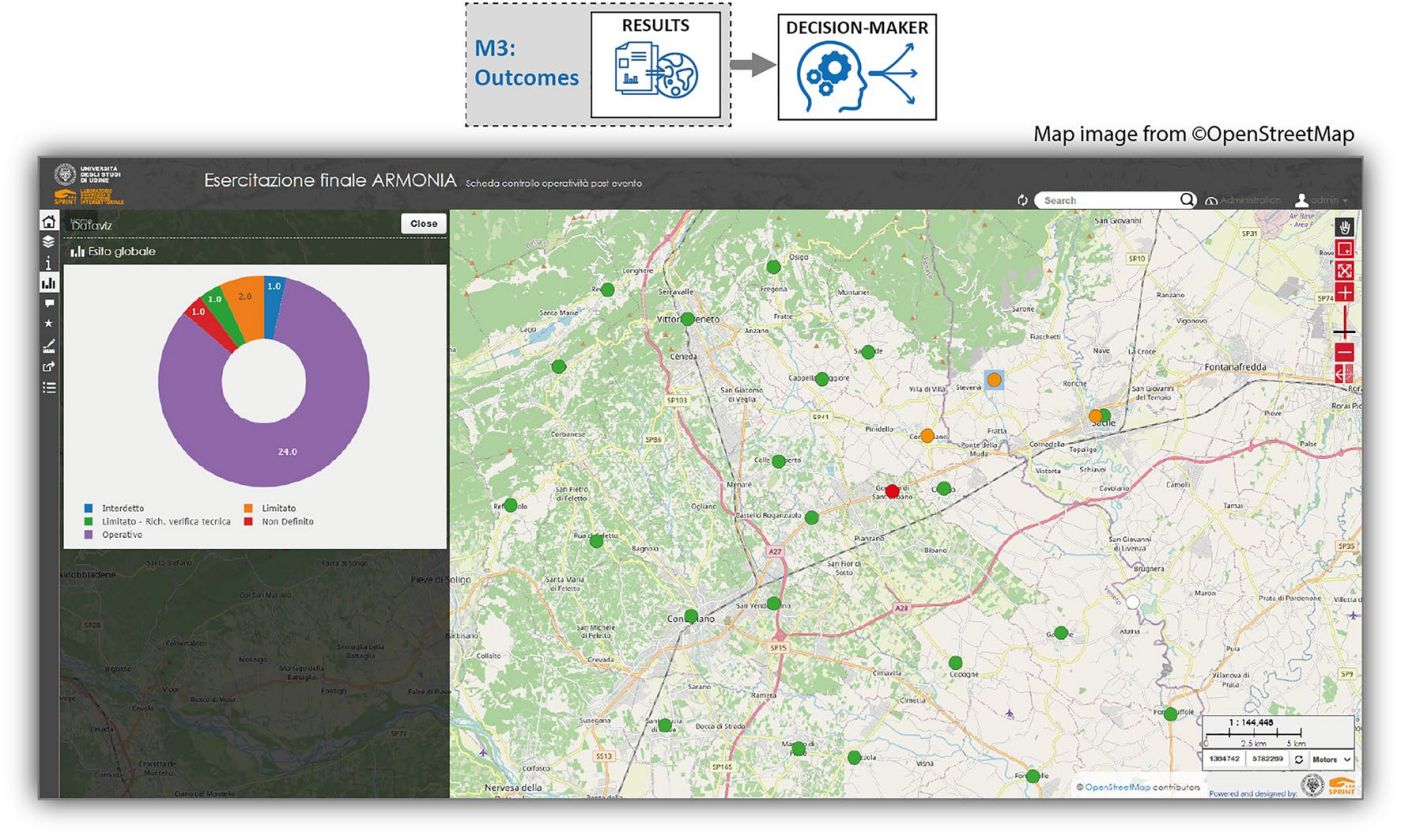
Example of outcomes obtained with the M3 SPRINT-Engine module: web map created in real-time with the outcomes of the COPEv form applied during a real-scale exercise in the northeast of Italy (map image from ©OpenStreetMap; Web Map Service: LizMap^15^).

The *outcomes* module allows to display the survey outcomes in real-time in web-maps; Fig. 6 shows an example of a web-map created during a real-scale exercise. These maps can be viewed and used in emergency rooms to depict the situation after the event and support the decision-making process^6^.

The COPEv form developed through the SPRINT-Engine has become an integral part of the procedures of the Regional Civil Protection of Friuli Venezia Giulia system. It has been adopted by regional municipalities and has been the subject of multiple real-scale experiments and exercises, demonstrating its usefulness. During post-application debriefings, it emerged that this tool provides valuable support to technicians called upon to carry out initial inspections of public buildings following a seismic event, as well as it expedites the overall recognition of the scenario for the surveyed area.

### RSP projects

The SPRINT-Lab researchers collaborate with PCR-FVG during the response phase after disasters. In particular, the researchers at the SPRINT-Lab used the SPRINT-Engine to develop a tool for trained volunteers of civil protection to quickly assess the damaged situation of buildings after a disaster. The tool developed for a very quick characterisation of the post-earthquake situation at a large scale is the RSP form. This is a simple form that supports the surveyor in the acquisition of the damage evidence after an earthquake. Six categories are considered, ranging from “complete collapse” to “no evidence of damage”. The form allows the damaged situation of a building to be assigned by simply clicking on the pictogram that summarises the situation; the option of using the app on a smartphone simplifies the survey. The coordinates of the device are automatically included in the survey, and if the accuracy is insufficient, the surveyor can change the position manually. In RSP projects, surveyors do not necessitate the validation of the data by a campaign manager and the SPRINT-Engine allows them to self-validate the surveys (this option is adopted when surveyors are also experts). The flexibility of the SPRINT-Engine also allows one to define particular subjects who have specific privileges in a campaign or project. The campaigns developed with the RPS forms can be used to collect information that is integrated with the outcomes of other forms, for an overall view in the PCR-FVG situation room.

The RSP form has allowed for the improvement in terms of expeditiousness and extension of the rapid assessment of the impact of a low or moderate-intensity seismic event, thanks to the contribution of volunteers present throughout the entire region, who have been trained to the use of the form. The RSP form has been used during training activities to enhance the preparedness of PCR-FVG volunteers. Additionally, it has been employed following small-magnitude earthquakes that have taken place within the Friuli Venezia Giulia Region’s territory, permitting a very rapid countercheck of the impact on the affected area.

### VISIVIA project

The *form builder* module of the SPRINT-Engine allows for the creation of complicated survey forms. This is the case of the forms developed in the framework of the VISIVIA project, established between SPRINT-Lab researchers and ANSFISA. The project is currently under development and initial experiments have been conducted. In VISIVIA, the SPRINT-Engine is used to create data acquisition and evaluation forms to support the inspection activities of the road network, comprising the connected infrastructures (e.g., bridges and tunnels). The possibility to customise the elements of the survey form through JavaScript allowed for the definition of a form that simplified the activities of a surveyor, as highlighted during a real-scale test. Three specific forms have been designed for roads, bridges, and tunnels. In the post-survey processing, the *outcome* module permits the connection of the information acquired using the three independent forms, and to group it on the basis of geographical information and of data included in the forms. This allows the creation of maps and dashboards for supporting decision-makers. From the web maps, it is possible to access the reports automatically created, as well as to go back to survey data and photos. The outcomes of the VISIVIA methodology are visualized in the ANSFISA operative room to provide decision-makers with an overview of the assessed situation.

## Final considerations

IT data collection tools are nowadays essential tools for collecting on-field data and supporting disaster management. However, disasters are usually characterised by great uncertainty, and this aspect often affects data collection tools, which should take into account the potential need for a real-time adaptation, considering the specific requirements that may arise in each context. If the assessment required data can change almost dynamically, it is important to ensure that any surveyor who may already be deployed in the field can use the updated versions of the data collection form to conduct the surveys. In addition, it is also important that the surveyors are trained to do the surveys, and in most cases, training is more successful if real-case examples are provided.

To address these issues, the Authors have designed and developed the SPRINT-Engine for the specific needs of SPRINT-Lab projects and activities to support institutions involved in different phases of DMC. The SPRINT-Engine is a customisable IT tool that allows the development of survey forms with integrated data elaboration, in an easy way and with IT basic knowledge. With the SPRINT-Engine it is possible to easily create and modify data collection forms and implement algorithms to create automated outcomes that support the decision-making process in all main phases of the DMC. The forms can be created or modified in real-time by a form builder manager in a management room (usually working remotely). When an online connection is available, the updated survey forms are shared with the surveyors. Then the surveyors can use the data collection application offline, which is essential in all situations where an internet connection is not available (e.g., in rural areas, or after a catastrophic event when the network is down). Furthermore, the data acquired by the surveyors enables the automatic reconstruction of the comprehensive overview of the situation in the control room.

In addition, the SPRINT-Engine can also be used as a capacity building tool in two ways. On the one hand, it allows the creation of test campaigns to train users in the use of the app and to understand how different assignments could affect the outcomes. On the other hand, the outcomes of the SPRINT-Engine can be used to create knowledge transfer tools, such as albums showing real cases from past projects, or case tests to show decision makers how the results can support them in the decision-making process.

The possibility of making prompt changes has allowed for a progressive improvement of the SPRINT-Engine, taking into account the feedback received from debriefings. Future applications will allow the SPRINT-Engine to be further improved using this logic of participative development and finalization with users.

## Data Availability

The datasets generated and analysed during the current study are not publicly available due to sensitivity of information but are available from the corresponding author on reasonable request. All the maps and tools illustrated in the paper were created using open-source software QGIS Server 3.22, LizMap, and Piwigo 13.8.0. URL links to open-source software: QGIS Server 3.22: https://docs.qgis.org/3.22/en/docs/server_manual/index.html; LizMap v.3.6 plugin: https://docs.lizmap.com/current/en/; Piwigo 13.8.0: https://piwigo.org/.
